# Quality and Reliability of Lung Cancer Treatment Short Videos on Chinese Social Media: A Cross‐Sectional Analysis for Cancer Education Improvement (January 1, 2020–October 30, 2025)

**DOI:** 10.1111/1759-7714.70273

**Published:** 2026-04-16

**Authors:** Zengrui Wang, Zhuang Luo, Le Wang, Jiefu Tang, Zhi Zhang, Xinli Fan

**Affiliations:** ^1^ Kunming Medical University Kunming China

**Keywords:** health education, lung cancer, social media, TikTok, video quality

## Abstract

**Background:**

Short‐form videos have become the common source of cancer information for Chinese patients and caregivers. We evaluated the content, quality, and reliability of lung cancer treatment videos on TikTok, Bilibili, and Kwai and generated evidence‐based recommendations for cancer health education.

**Methods:**

We conducted a cross‐sectional study of lung cancer treatment short videos on TikTok, Bilibili, and Kwai. The top 200 most‐liked videos per platform posted between January 1, 2020 and October 30, 2025, were retrieved on November 1, 2025. After screening, 300 videos (100 per platform) were analyzed. Two oncologists rated quality using GQS (1–5) and DISCERN (1–5); creator identity was classified. Comment sentiment (SnowNLP) and engagement metrics were analyzed.

**Results:**

TikTok had the highest engagement and quality (GQS 3.0, DISCERN 3.0) versus Bilibili/Kwai (2.0) (*p* < 0.001). Professionals achieved the highest quality (GQS 3.0) versus institutions (1.0) (*p* < 0.001). However, absolute quality was low across all platforms: only 6% of videos met high‐quality criteria (GQS ≥ 4), and 5% met DISCERN ≥ 4. Engagement showed a weak negative correlation with quality (*ρ* = −0.13 to −0.21).

**Conclusions:**

Overall quality is low; professional content is more reliable but less viral. Embedding quality indicators in algorithms and promoting certified creators could improve patient cancer education.

## Graphical abstract

Short videos on Chinese social media have become a primary source of lung cancer information for patients and caregivers. This cross‐sectional study analyzed 300 lung cancer treatment videos from TikTok, Bilibili, and Kwai (2020–2025). Overall quality was alarmingly low: only 6% of videos met high‐quality criteria (GQS ≥ 4), and 5% met DISCERN ≥ 4. TikTok had the highest quality (median GQS 3.0), but engagement metrics correlated negatively with quality. Professional medical personnel produced the most reliable content, yet their videos were less viral than lower‐quality ones. Sentiment analysis showed 46% positive and 12% negative comments, with negative feedback often questioning the creator credentials. Embedding quality indicators into recommendation algorithms and promoting verified oncology creators are urgently needed to transform scrolling into valuable patient education.

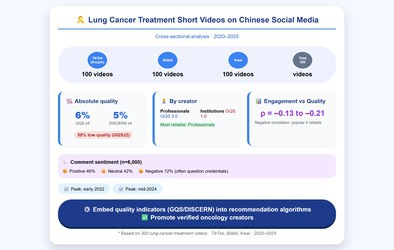

## 1 Introduction

Lung cancer remains the leading cause of cancer mortality in China, with approximately 820 000 new cases annually [1]. Between 2020 and 2025, short‐form video platforms such as TikTok (Douyin), Bilibili, and Kwai have become the common source of cancer information for patients and caregivers [2, 3, 4, 5]. However, the brevity and entertainment‐oriented algorithms of these platforms may facilitate misinformation [6, 7, 8]. Previous international studies have examined YouTube or TikTok content in breast or skin cancer [9, 10, 11]; large‐scale cross‐platform comparisons focused on lung cancer treatment videos in the Chinese context are lacking [12, 13, 14]. We conducted a comprehensive content analysis of videos posted between January 1, 2020 and October 30, 2025, to evaluate quality and reliability across the three most popular Chinese platforms and to propose actionable recommendations for cancer health education [15, 16, 17, 18]. We hypothesized that (1) the overall quality of lung cancer treatment short videos is low, (2) content uploaded by professional medical personnel achieves higher quality scores than that from other creator types, and (3) user engagement metrics (likes, shares, comments) are not positively correlated with video quality.

## 2 Methods

### 2.1 Study Design and Reporting

We followed the STROBE checklist for cross‐sectional studies (checklist in File S1) [19]. Ethics approval was waived because only publicly available data were analyzed.

### 2.2 Search Strategy and Sampling

We searched TikTok (Douyin), Bilibili, and Kwai on November 1, 2025 using 12 Chinese‐language keywords related to lung cancer treatment: “肺癌治疗” (lung cancer treatment), “肺癌靶向治疗” (lung cancer targeted therapy), “肺癌免疫治疗” (lung cancer immunotherapy), “肺癌化疗” (lung cancer chemotherapy), “肺癌手术” (lung cancer surgery), “肺癌放疗” (lung cancer radiotherapy), “肺癌中医治疗” (TCM lung cancer treatment), “肺癌康复” (lung cancer rehabilitation), “肺癌副作用” (lung cancer side effects), “肺癌饮食” (lung cancer diet), “肺癌专家” (lung cancer specialist), and “肺癌科普” (lung cancer education). For each platform, we applied the internal “sort by likes” filter to retrieve the most popular videos. The top 200 videos per platform posted between January 1, 2020 and October 30, 2025, were screen‐recorded and saved for further screening [10, 20, 21]. To minimize personalization bias, we logged out of personal accounts, cleared browser cache, and used incognito/private mode before each search. The region was set to Mainland China, and the language was restricted to Chinese.

**FIGURE 1:**
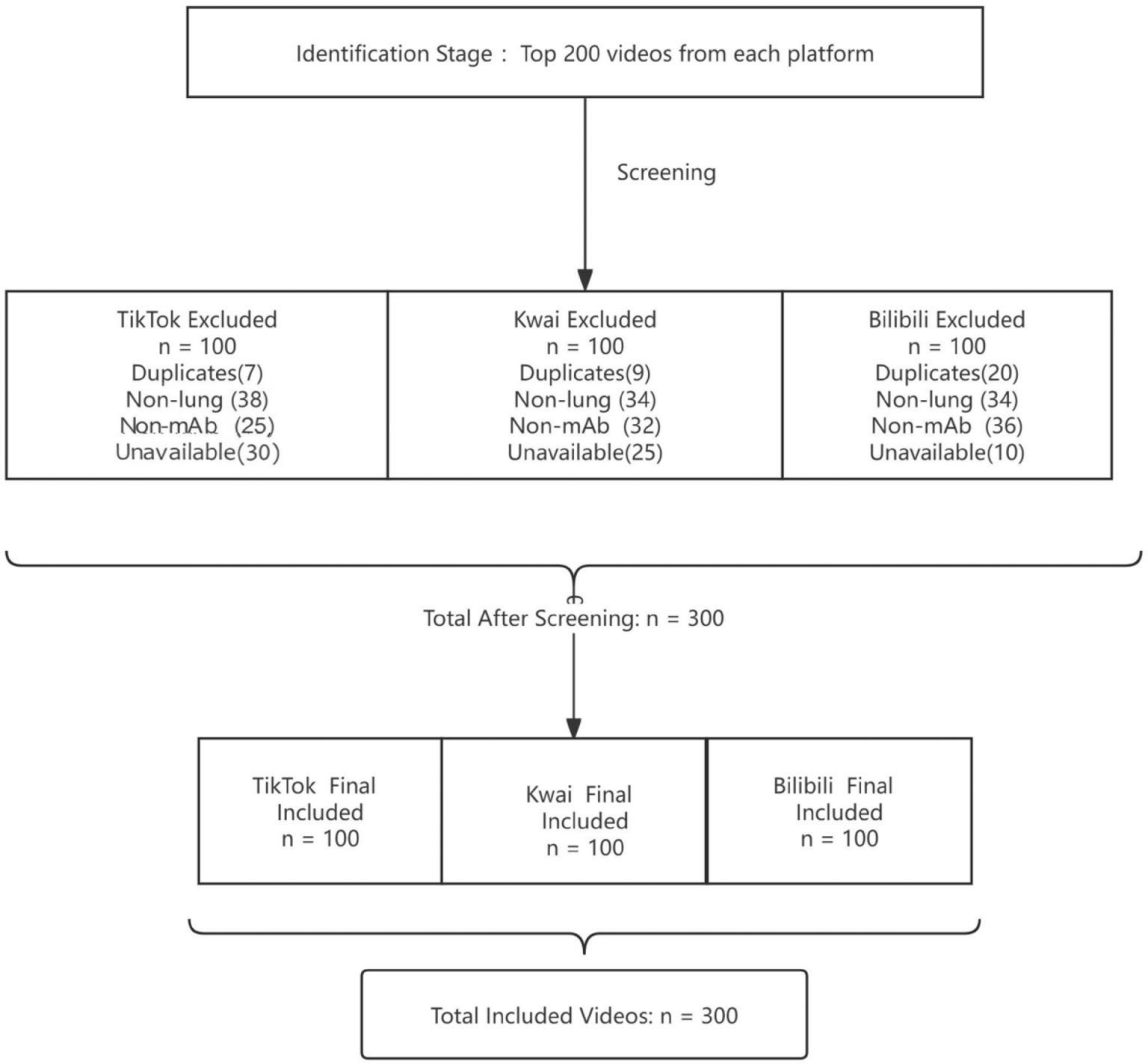
Flow diagram of video selection process (January 1, 2020–October 30, 2025).

After deduplication and eligibility screening, 100 videos per platform were included in the final analysis (see Figure 1 for flow diagram).

### 2.3 Eligibility Criteria

Inclusion: (i) Chinese language, (ii) visual–auditory content primarily related to lung cancer treatment, and (iii) duration ≥ 5 s. Exclusion: duplicates, live‐stream replays, advertisements, or videos deleted within 2 weeks of capture [22, 23, 24].

### 2.4 Sample Size Justification

We set a target sample size of 100 videos per platform based on prior studies [5, 6]. After applying the inclusion and exclusion criteria, exactly 100 videos per platform remained eligible; thus, no further random selection was needed. This coincidence reflects the consistent application of screening criteria across platforms, which demonstrated sufficient power to detect between‐group differences in GQS scores (effect size = 0.4, *α* = 0.05, *β* = 0.20). This sample size also aligns with common practice in cross‐platform health information studies and ensures the feasibility of manual coding and quality assessment.

### 2.5 Data Extraction

Two authors independently extracted: video URL, duration, likes, collections, comments, shares, upload date, and creator identity. Creator identity was classified as (a) professional medical personnel (verified physician or hospital), (b) nonprofessional medical personnel (nurse, technician), (c) institution (hospital or corporate account), or (d) individual (layperson) [25, 26, 27]. Disagreements were resolved by discussion.

We intentionally sampled the top 200 most‐liked videos per platform to reflect content with the highest visibility and user engagement, as these are most likely to influence patient education. However, this approach may introduce selection bias toward popular content, potentially excluding high‐quality videos with low‐engagement metrics.

After applying the inclusion and exclusion criteria, exactly 100 videos per platform remained eligible for analysis. This coincidental outcome reflects the consistent application of our screening criteria across platforms and the similar proportion of relevant content meeting inclusion standards.

### 2.6 Content Classification

We coded the main topic into one of five mutually exclusive categories: etiology, clinical manifestations, diagnosis, treatment, and prognosis [28, 29].

### 2.7 Quality Assessment

Videos were rated independently by two oncologists (Z.W. and L.W.) using (i) the Global Quality Score (GQS; 1 = *poor*, 5 = *excellent*) [30] and (ii) the DISCERN instrument (16 items, converted to a 5‐point scale, with higher scores indicating better quality) [31]. Both tools have been validated for short‐form video evaluation [32, 33]. Before rating, both raters underwent a 1‐h training session using five pilot videos that were not included in the final sample. Inter‐rater reliability was assessed using Cohen's weighted kappa (*κ*) for both GQS and the converted 5‐point DISCERN scores, as both instruments yield ordinal data. The weighted *κ* for GQS was 0.82 (95% CI: 0.76–0.88), and for DISCERN was 0.79 (95% CI: 0.72–0.85), indicating substantial to almost perfect agreement [34]. Two oncologists independently rated all videos. The average of their scores was used for all analyses. In cases where the initial scores differed by ≥ 2 points (indicating substantial disagreement), the two raters discussed the video to reach consensus; if consensus could not be achieved, a third senior oncologist (Z.L.) was consulted for final adjudication. This approach ensured data quality while minimizing subjective bias.

### 2.8 Definition of Quality Thresholds

Based on previously established criteria in short‐form video content analysis [32, 33], we defined videos with GQS ≥ 4 and DISCERN ≥ 4 as “high quality.” Videos scoring 3 were considered “*moderate quality*,” and those scoring ≤ 2 were classified as “*low quality*.” This classification allows for both absolute quality assessment and comparative analyses across platforms and creator types.

### 2.9 Sentiment Analysis

We randomly sampled 20% of user comments (≈6000) under the 300 videos and classified sentiment with SnowNLP (accuracy 0.83) into positive, neutral, or negative [4, 11].

### 2.10 Cross‐Platform Comparability

We acknowledge that TikTok, Bilibili, and Kwai differ in user demographics, video length limits, content formats, and recommendation algorithms. However, all three platforms are among the most widely used short‐video platforms in China and serve as primary sources of health information for patients and caregivers [2, 3, 4]. Our primary aim was to describe and compare the quality and reliability of lung cancer treatment content across these platforms from a user's perspective, rather than to establish causal relationships. To account for platform‐specific characteristics, we report all outcomes stratified by platform and avoid aggregating scores into a single composite measure. Multivariable adjustment was not performed due to the descriptive nature of the study, but we highlight platform‐level differences in the interpretation of findings.

### 2.11 Statistical Analysis

We used R 4.3.3 (Zstats 1.0 package) [35]. Continuous variables are presented as median (Q1, Q3) and categorical variables as *n* (%). Between‐platform comparisons were performed with Kruskal–Wallis tests (continuous) or *χ* ^2^ tests (categorical). Correlation between engagement metrics and quality scores was assessed with Spearman's *ρ* [36, 37, 38]. Two‐tailed *p* < 0.05 was considered significant.

### 2.12 Ethics Approval

This study used publicly available data from social media platforms and did not involve human participants.

## 3 Results

Of 600 videos retrieved (January 1, 2020–October 30, 2025), 300 were finally analyzed (100 per platform). Figure 1 summarizes the selection process.

### 3.1 General Characteristics and Engagement

Table 1 presents median (Q1, Q3) values for duration, likes, collections, comments, and shares. TikTok achieved the highest engagement on all metrics. Kruskal–Wallis tests revealed significant differences across platforms for all engagement metrics (all *p* < 0.001). Post hoc pairwise comparisons using Dunn's test with Bonferroni correction showed that TikTok significantly outperformed both Bilibili and Kwai on likes, collections, comments, and shares (all adjusted *p* < 0.001), while differences between Bilibili and Kwai were not statistically significant for most metrics (Table 1). Median video length differed significantly: Kwai 31 s (7–86), TikTok 147 s (94–180), and Bilibili 189 s (81–487) (*p* < 0.001).

**TABLE 1:** General characteristics and engagement metrics of videos by platform (*n* = 300).

Variables	TikTok (*n* = 100)	Bilibili (*n* = 100)	Kwai (*n* = 100)	*p*
General information video				
Length(s), *M* (Q1, Q3)	147.00 (93.75, 180.00)	189.00 (80.50, 486.50)	31.00 (7.00, 86.00)	< 0.001
Likes, *M* (Q1, Q3)	670.00 (361.00, 2092.50)	20.50 (4.00, 160.00)	493.50 (90.75, 1746.75)	< 0.001
Collections, *M* (Q1, Q3)	307.00 (116.00, 967.50)	22.50 (4.00, 160.00)	234.50 (27.00, 773.00)	< 0.001
Comments, *M* (Q1, Q3)	86.50 (29.75, 245.75)	2.00 (0.00, 24.00)	57.00 (12.00, 352.25)	< 0.001
Shares, *M* (Q1, Q3) video	138.50 (56.00, 473.75)	5.50 (1.00, 60.00)	301.50 (40.75, 1118.75)	< 0.001
Content				
Etiology	1	0	19	
Clinical manifestation	3	1	52	
Diagnosis	2	0	11	
Treatment	100	100	97	
Prognosis video quality	3	0	7	
GQS score, *M* (Q1, Q3)	3.00 (2.00, 3.00)	2.00 (2.00, 3.00)	2.00 (1.00, 2.00)	< 001
mDISCERN score, *M* (Q1, Q3)	3.00 (2.00, 3.00)	2.00 (2.00, 2.00)	2.00 (1.00, 2.00)	< 0.001

*Note:* Data are presented as median (Q1, Q3).

Abbreviations: *M*, median; Q1, 25th percentile; Q3, 75th percentile.

### 3.2 Quality Scores by Platform

Median GQS and DISCERN were highest on TikTok (3.0 for both), followed by Bilibili (2.0) and Kwai (2.0) (Kruskal–Wallis test, *p* < 0.001) (Table 1). In terms of absolute quality, only 6% (18/300) of videos achieved GQS ≥ 4 (high quality), and 5% (15/300) achieved DISCERN ≥ 4 (high quality). The majority of videos were of low quality (GQS ≤ 2: 58%; DISCERN ≤ 2: 62%), indicating that most lung cancer treatment short videos fail to meet basic quality standards.

### 3.3 Content Domains

**FIGURE 2:**
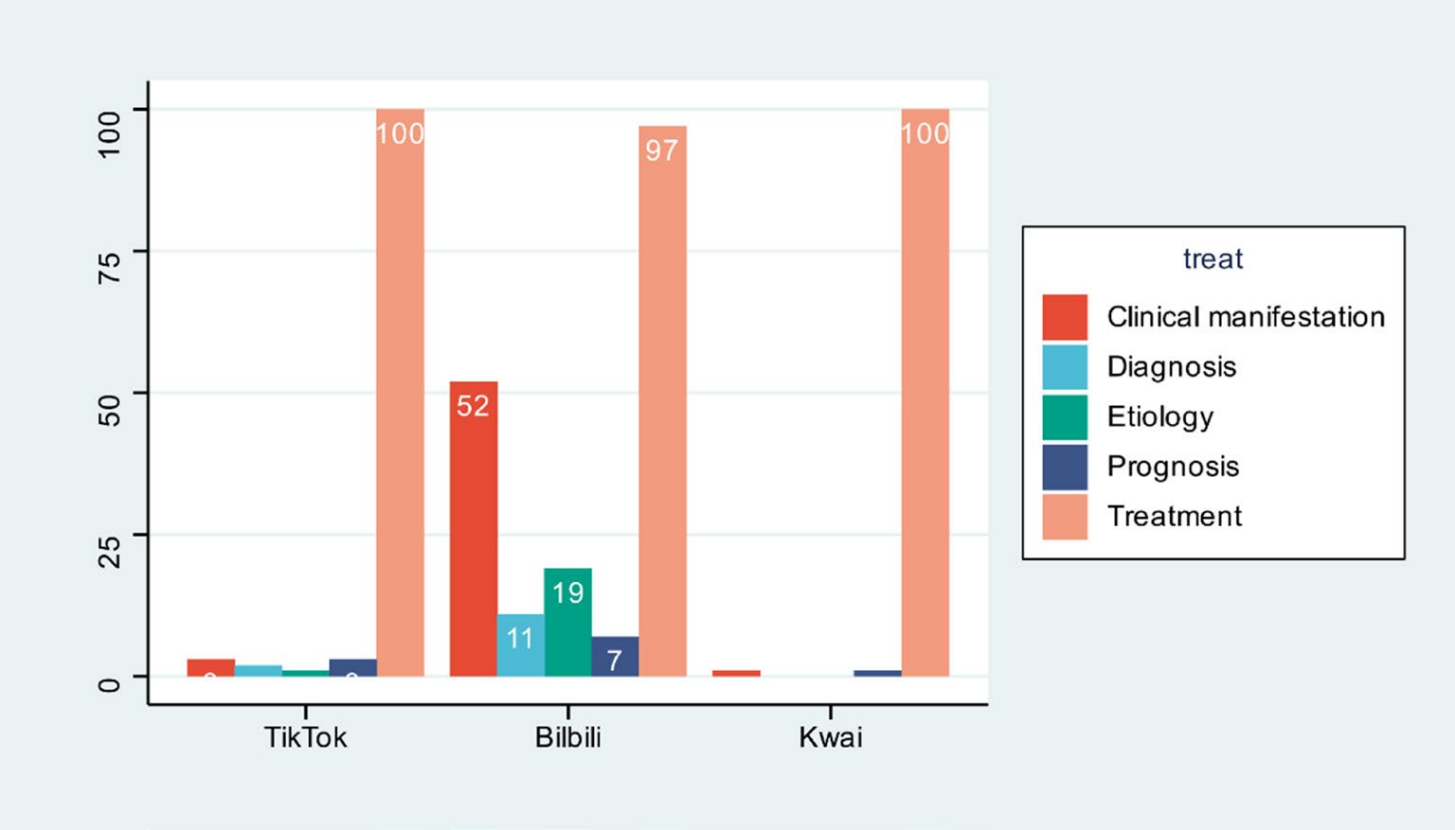
Content topic distribution across the three platforms (%). Percentages may sum to > 100% because a single video could be classified into multiple content categories.

Treatment dominated all platforms (TikTok 100%, Bilibili 100%, Kwai 97%). Kwai contained markedly more “clinical manifestations” (52%) and “etiology” videos (19%) than the other two sites (*χ* ^2^ = 126.4, *p* < 0.001) (Figure 2).

**FIGURE 3:**
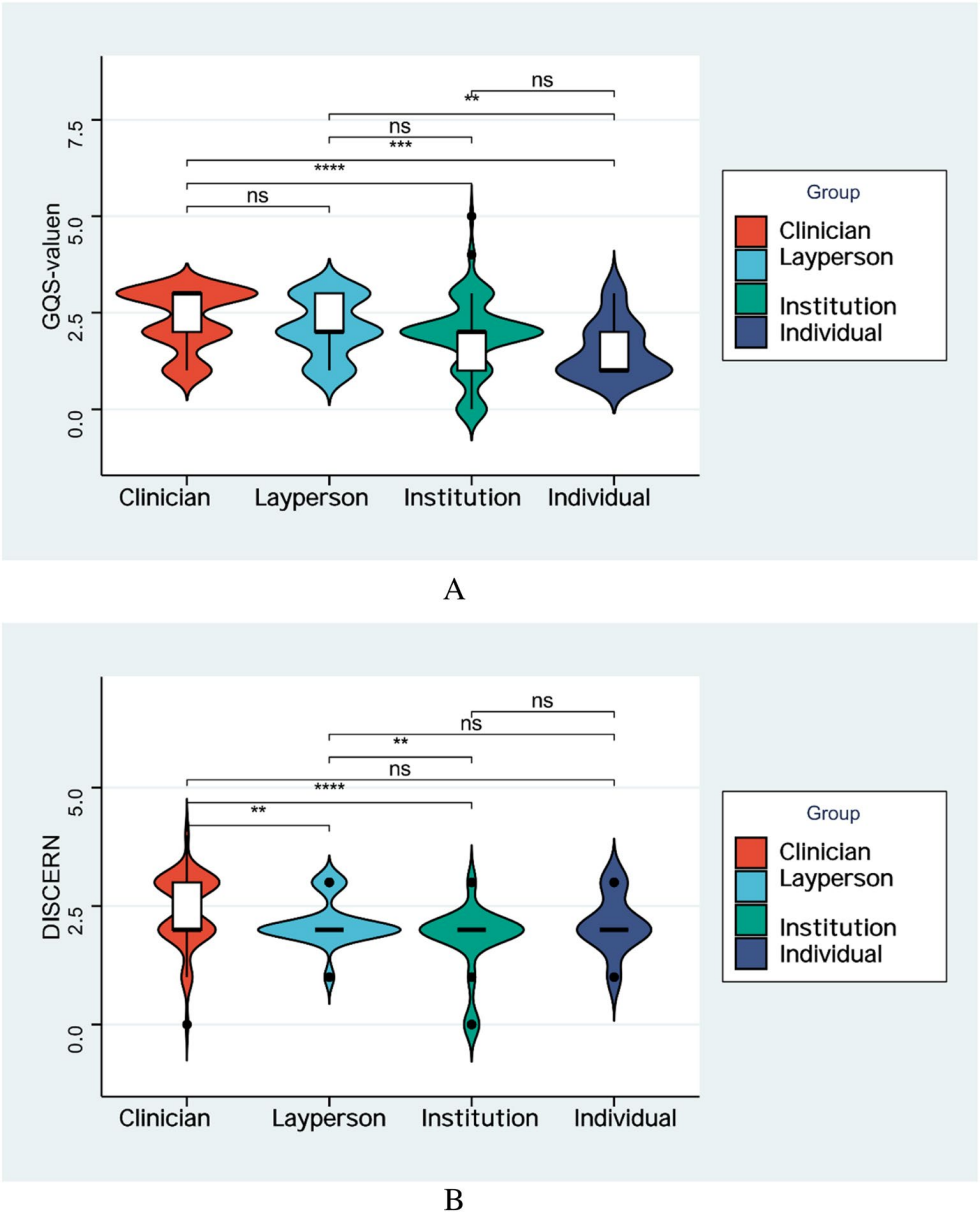
Box plots of GQS and DISCERN scores by creator identity.

Figure 3 presents quality scores stratified by creator identity. (A) Descriptive statistics for GQS. Median scores were identical across groups (2.7), with interquartile ranges (Q1–Q3) of approximately 2.6–2.9, indicating uniformly low quality. Professionals (*n* = 125) and individuals (*n* = 99) were the most common creators, while institutions were rare (*n* = 17). Maximum scores of 5.0 suggest occasional high‐quality videos. (B) Standardized DISCERN scores (0–1 scale) with group comparisons. Compared to professionals, laypersons and institutions showed no significant difference (ns), whereas individual creators scored significantly lower (**p < 0.01). Overall, DISCERN scores remained low across all creator types, with minimal clinically meaningful differences.

### 3.4 Quality by Creator Identity

**FIGURE 4:**
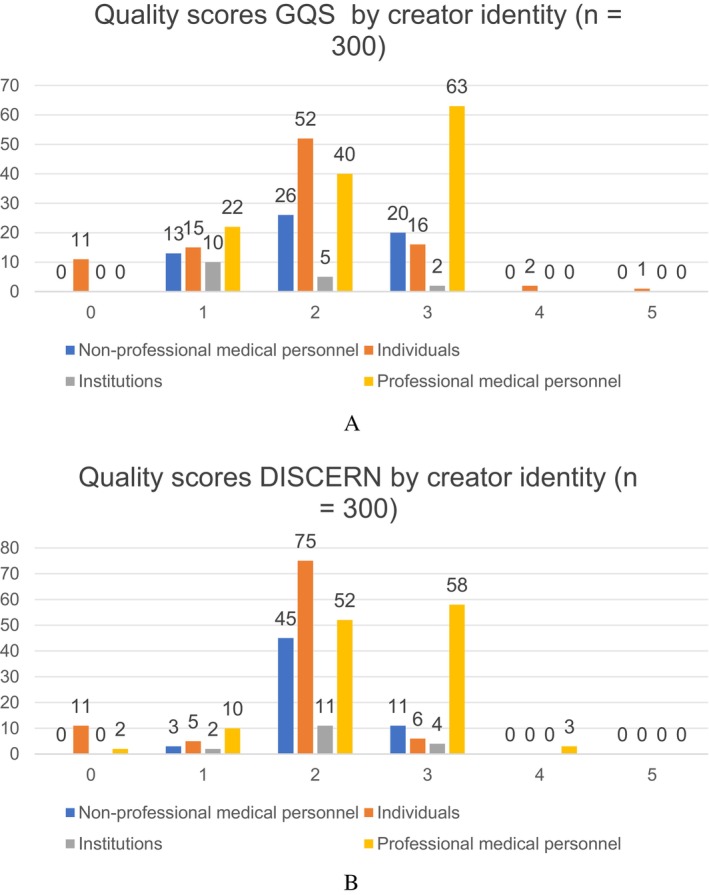
Distribution of GQS and DISCERN scores by creator identity.

Professional medical personnel achieved the highest median GQS (3.0) and DISCERN (3.0), whereas institution‐generated videos had the lowest (1.0 for both) (Kruskal–Wallis test, *p* < 0.001). Figure 4 presents the distribution of GQS and DISCERN scores by creator identity. Details are summarized in Table 2.

**TABLE 2:** Quality scores (GQS and DISCERN) by creator identity (*n* = 300).

Variables	Total (*n* = 300)	Nonprofessional medical personnel (*n* = 59)	Individuals (*n* = 97)	Institutions (*n* = 19)	Professional medical personnel (*n* = 125)	*p*
Video length(s), *M* (Q1, Q3)	108.50 (45.25, 189.75)	147.00 (67.50, 240.50)	131.00 (63.00, 421.00)	6.00 (4.00, 52.00)	90.00 (45.00, 151.00)	< 0.001
Likes, *M* (Q1, Q3)	365.50 (33.00, 1395.75)	654.00 (74.50, 2723.50)	52.00 (5.00, 368.00)	287.00 (87.00, 994.00)	534.00 (147.00, 1395.00)	< 0.001
Collections, *M* (Q1, Q3)	140.00 (18.25, 650.75)	269.00 (23.50, 1395.50)	41.00 (4.00, 238.00)	65.00 (15.00, 452.00)	251.00 (51.00, 769.00)	< 0.001
Comments, *M* (Q1, Q3)	35.00 (4.00, 199.75)	80.00 (13.50, 429.00)	4.00 (1.00, 96.00)	14.00 (5.00, 78.00)	62.00 (13.00, 204.00)	< 0.001
Shares, *M* (Q1, Q3)	100.00 (8.00, 569.50)	189.00 (20.50, 695.50)	10.00 (3.00, 168.00)	147.00 (40.00, 596.00)	132.00 (38.00, 670.00)	< 0.001
GQS, *M* (Q1, Q3)	2.00 (2.00, 3.00)	2.00 (2.00, 3.00)	2.00 (1.00, 2.00)	1.00 (1.00, 2.00)	3.00 (2.00, 3.00)	< 0.001
SUM, *M* (Q1, Q3)	2.00 (2.00, 3.00)	2.00 (2.00, 2.00)	2.00 (2.00, 2.00)	2.00 (2.00, 2.00)	2.00 (2.00, 3.00)	< 0.001

*Note:* Data are presented as median (Q1, Q3). *p* value from the Kruskal–Wallis test comparing GQS across creator identity groups.

Abbreviations: *M*, median; Q1, 25th percentile; Q3, 75th percentile.

### 3.5 Correlation Analysis

**FIGURE 5:**
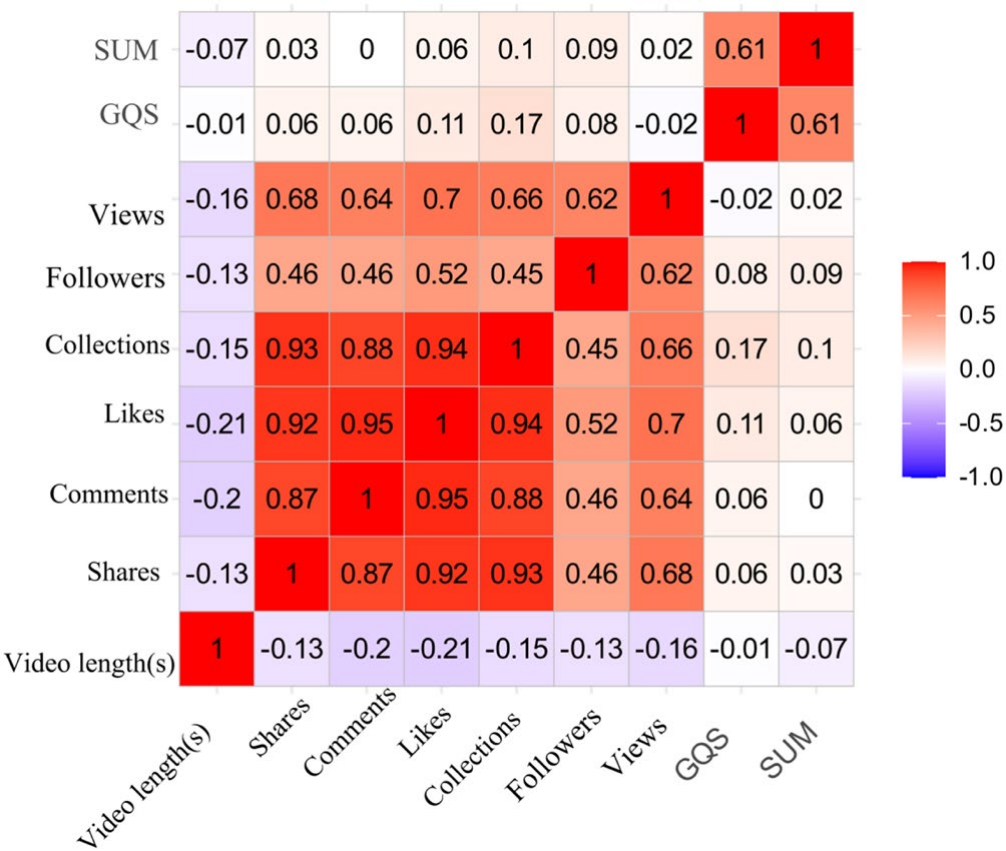
Heat map of Spearman correlations between engagement metrics and quality scores.

Likes, collections, comments, and shares were strongly intercorrelated (*ρ* = 0.87–0.95, all *p* < 0.001), but each engagement metric was weakly negatively correlated with both GQS and DISCERN (*ρ* = −0.13 to −0.21, *p* < 0.05) (Figure 5).

### 3.6 Creator Identity Distribution

**FIGURE 6:**
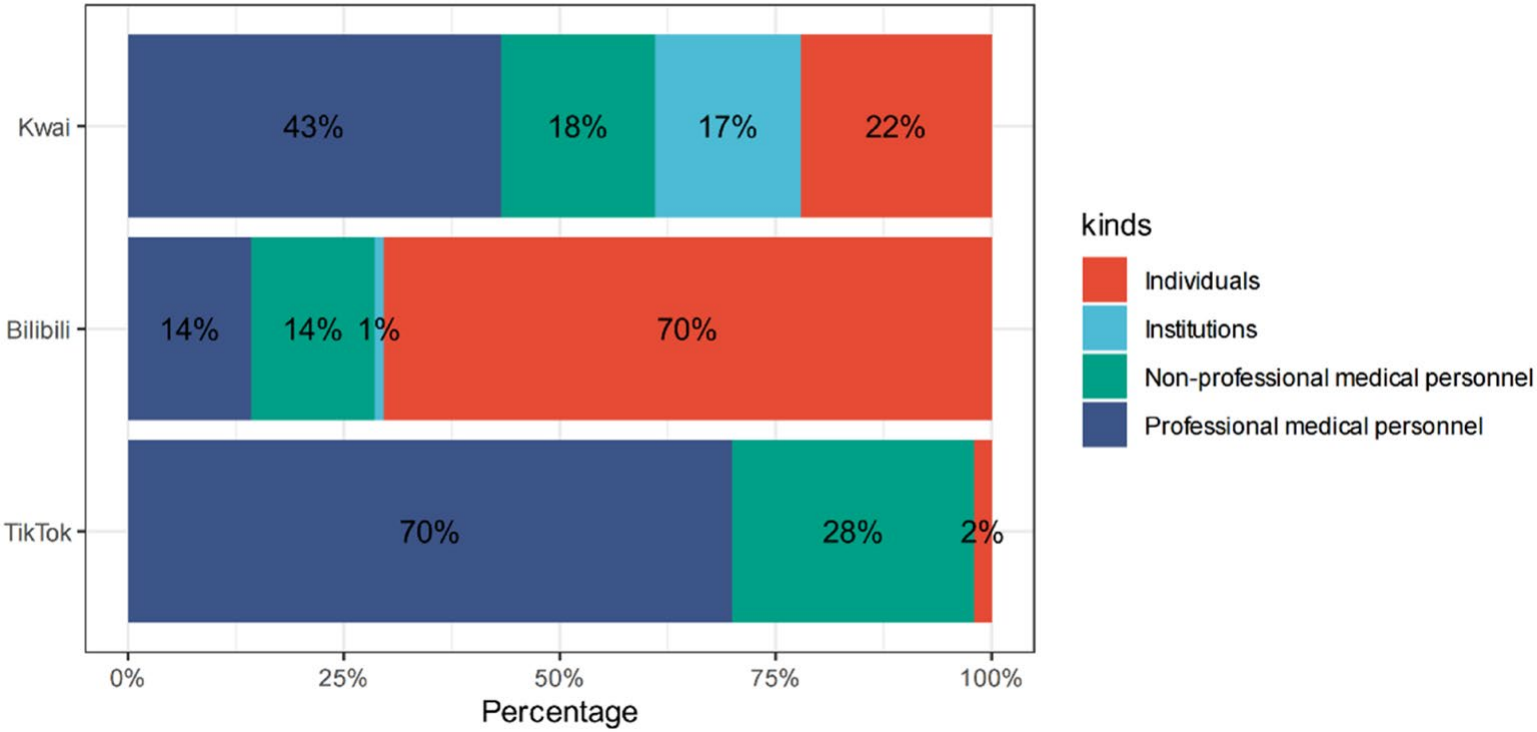
Stacked bar chart of creator identity by platform (*n* = 300).

Across 300 videos, 41.7% were uploaded by professional medical personnel, 32.6% by individuals, 19.7% by nonprofessional medical staff, and 5.7% by institutions (Figure 6). Platform‐wise, TikTok was dominated by professionals (70%), Bilibili by individuals (70%), whereas Kwai showed a more balanced mix (Figure 6).

### 3.7 Annual Upload Trend

**FIGURE 7:**
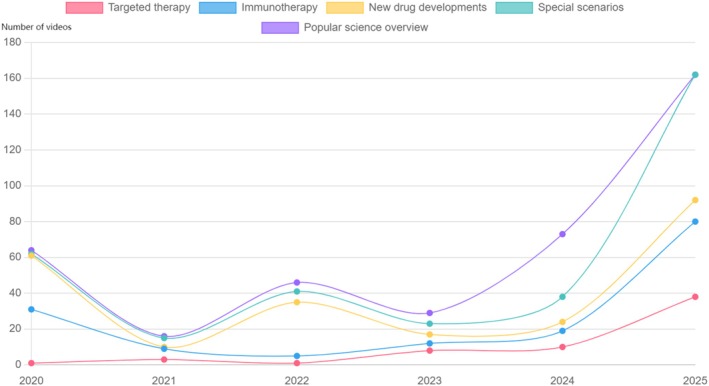
Annual number of lung cancer treatment short videos uploaded across TikTok, Bilibili, and Kwai combined.

Stacked area analysis showed two clear peaks: early 2022 (National Cancer Awareness Week) and mid‐2024 (new drug launch campaign), with TikTok contributing the largest annual share throughout 2020–2025 (Figure 7). Two peaks are observed: early 2022 (National Cancer Awareness Week) and mid‐2024 (new drug launch campaign).

### 3.8 Comment Sentiment

**FIGURE 8:**
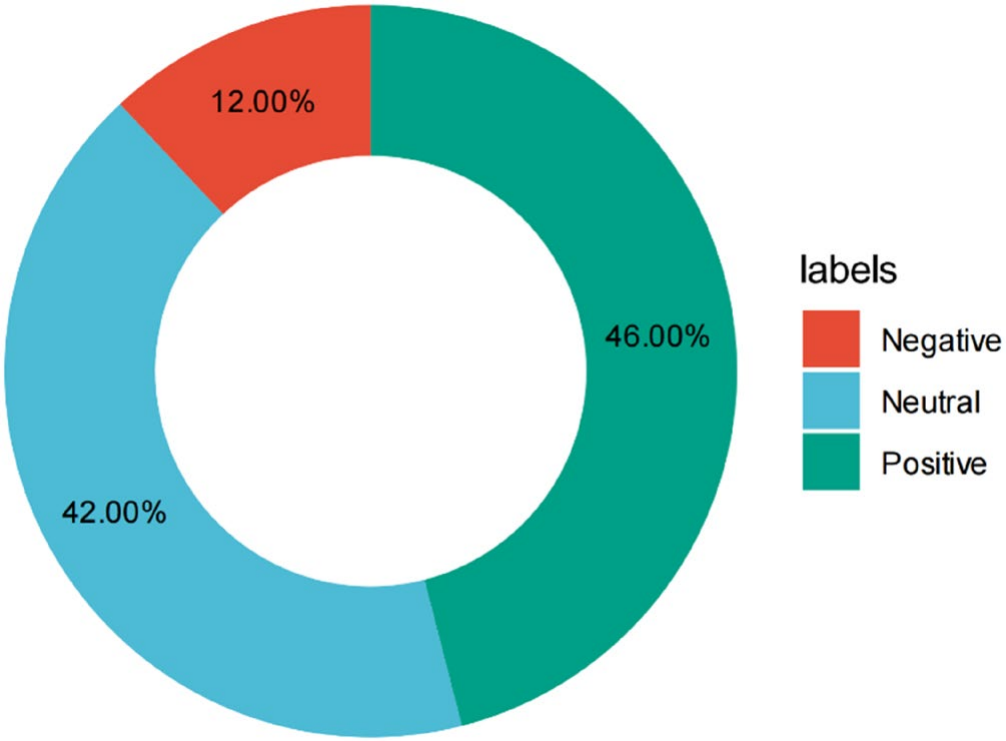
Sentiment distribution of user comments (*n* = 6000 randomly sampled).

SnowNLP classification of 6000 randomly sampled comments revealed 46% positive, 42% neutral, and 12% negative sentiment; negative comments frequently questioned creator credentials or underreported side‐effects (Figure 8).

This is a cross‐platform, cross‐temporal analysis of lung cancer treatment short videos on Chinese social media, comparing TikTok, Bilibili, and Kwai using validated instruments (GQS and DISCERN) and sentiment analysis (SnowNLP) [39, 40, 41]. We found that (i) overall quality is low—only one in 20 videos meets high‐quality criteria, (ii) professional medical creators significantly outperform institutions or laypersons, (iii) high user engagement does not equate to high quality, and (iv) audience sentiment is predominantly positive/neutral, yet 12% negative comments often question creator credentials or underreported side‐effects [42].

### 3.9 Comparison With Prior Work

Our GQS median (2.0–3.0) aligns with recent TikTok breast cancer (2.5) and YouTube lung cancer (2.0) reports, but is lower than physician‐created prostate cancer videos on TikTok (3.4). The weak negative correlation between popularity and quality observed in our study aligns with previous reports, suggesting that highly engaged content is not necessarily reliable. This finding highlights the potential value of incorporating quality indicators into recommendation algorithms, although further research is needed to establish causality and assess technical feasibility.

Implications for cancer education:

1. *For clinicians*: Verified oncologists should proactively produce 60–90 s evidence‐based videos that address treatment side‐effects and prognosis, as these topics accounted for < 5% in our sample.
2. *For platform designers*: As a conceptual consideration, integrating quality indicators such as DISCERN or GQS into recommendation algorithms might help prioritize reliable content, although the technical feasibility and real‐world impact require further investigation. Even a modest weighting could potentially reduce the visibility of low‐quality videos without compromising user engagement.
3. *For patient educators*: Hospitals can create QR‐code “white‐lists” of high‐scoring videos and integrate them into discharge handouts, ensuring that patients watch reliable information during perioperative periods.
4. *For policymakers*: A specialty‐specific certification (e.g., “Oncology Verified Creator”) akin to YouTube Health certification should be piloted on Chinese platforms.

### 3.10 Strengths and Limitations

We analyzed the largest sample of trending videos over 6 years and used validated quality tools. However, our sampling strategy focused exclusively on the top 200 most‐liked videos per platform, which may have excluded high‐quality but low‐engagement content. As a result, our findings may not be representative of the entire corpus of lung cancer treatment short videos. Although we included videos uploaded until October 30, 2025, data collection was performed on November 1, 2025, ensuring that all eligible videos within the study period were captured. However, videos uploaded after October 30, 2025, were not considered, which may slightly limit the timeliness of our findings. In addition, the platforms evaluated in this study differ substantially in audience demographics, video length restrictions, and algorithmic content delivery. These differences may influence both the type of content produced and the engagement patterns observed. Therefore, direct comparisons between platforms should be interpreted with caution. Despite these differences, our core findings—low overall quality, superior performance of professional creators, and a negative correlation between engagement and quality—were consistent across platforms, suggesting they may reflect broader trends in short‐form health content. In addition, we did not assess comment accuracy or viewer comprehension, and the cross‐sectional design precludes causal inferences. Future work should combine eye‐tracking with pre–post knowledge tests to evaluate real learning outcomes, and employ stratified sampling methods (e.g., based on upload date, creator type, or random sampling) to obtain a more comprehensive picture of content quality across platforms.

## 4 Conclusions

Among the most‐viewed lung cancer treatment short videos on Chinese social media, the content is more likely to entertain than to educate. Prioritizing content from certified medical professionals and considering the integration of quality metrics into platform algorithms may help transform scrolling time into valuable cancer learning opportunities.

## Author Contributions

**Zengrui Wang:** conceptualization, methodology, formal analysis, data curation, software, investigation, writing – original draft, writing – review and editing, visualization. Zengrui Wang is the sole first author of this manuscript. **Zhuang Luo:** conceptualization, supervision, writing – review and editing, investigation. **Le Wang:** investigation. **Jiefu Tang:** validation. **Zhi Zhang:** software. **Xinli Fan:** resources. All authors have read and agreed to the published version of the manuscript.

## Funding

This work was supported by the National Science and Technology Major Project “NoncommuniIcable Chronic Disease Prevention and Control Research” (Grant no. 2022535D01), Young and Middle‐Aged Leaders and Reserve Candidates of Kunming Medical University – “Cheng Feng” Talent Training Program, and Yunnan Xingdian Talent Support Program (Grant no. RLMY20220004).

## Conflicts of Interest

The authors declare no conflicts of interest.

## Supporting information


**DATA S1:** STROBE Statement—Checklist of items that should be included in reports of cross‐sectional studies.

## Data Availability

The data that support the findings of this study are available on request from the corresponding author. The data are not publicly available due to privacy or ethical restrictions.
